# Mixed kernel SVR addressing Parkinson’s progression from voice features

**DOI:** 10.1371/journal.pone.0275721

**Published:** 2022-10-07

**Authors:** Roberto Bárcenas, Ruth Fuentes-García, Lizbeth Naranjo

**Affiliations:** Departamento de Matemáticas, Facultad de Ciencias, Universidad Nacional Autónoma de México, Ciudad Universitaria, Mexico City, Mexico; Hefei University of Technology, CHINA

## Abstract

Parkinson’s Disease (PD) is a progressive neurodegenerative disease with multiple motor and non-motor characteristics. PD patients commonly face vocal impairments during the early stages of the disease. In this article, the aim is to explain the Unified Parkinson’s Disease Rating Scale (UPDRS) as a measure of the progression of Parkinson’s disease using a set of covariates obtained from voice signals. In particular, a Support Vector Regression (SVR) model based on a combination of kernel functions is introduced. Theoretically, this proposal, that relies on a mixed kernel (global and local) produces an admissible kernel function. The optimal fitting was obtained for the combination given by the product of radial and polynomial basis. Important results are the non-linear relationships inferred from the features to the response, as well as a considerable improvement in prediction performance metrics, when compared to other learning approaches. Furthermore, with knowledge on factors such as age and gender, it is possible to describe the dynamics of patients’ UPDRS from the data collected during their monitoring. In summary, these advances could expand learning processes and intelligent systems to assist in monitoring the evolution of Parkinson’s disease.

## 1 Introduction

Parkinson’s disease (PD) is a neurodegenerative disorder that affects body movement and occurs when neurons stop producing dopamine, a neurotransmitter existing in the brain and central nervous system. It is a progressive disease characterized by several motor symptoms, such as tremor, muscle stiffness, difficulty in performing voluntary movements, slowness or bradykinesia, postural instability, gait disturbance, and propensity to bend the trunk forward. These symptoms begin asymmetrically and gradually the opposite side is affected. The most frequent initial symptom is a rest tremor with a frequency of 4 to 6 cycles/second and, although it is the most visible manifestation, it is not the most disabling. PD is also associated with other non-motor dysfunctions reflected into cognitive alterations and, as the disease progresses, neuropsychiatric alterations [1].

The diagnosis of PD is clinical and is conducted based on the discovery of at least 2 or 3 of the following signs: tremor, bradykinesia, rigidity, alterations of postural reflexes, and the careful exclusion of secondary parkinsonisms or those associated with other neurological diseases. Multiple factors can produce this condition, but, in most cases, the direct cause that triggers it is unknown. Up to now, there is no cure for this disease. Since a wide spectrum of motor and non-motor symptoms characterizes PD, clinical assessment of these symptoms and signs is key to the proper control of the disease. The Hoehn-Yahr staging and the Unified Parkinson’s Disease Rating Scale (UPDRS) are measures used to assess the patient’s motor status and disease progression. They also act as a system of response to symptomatic treatment. The UPDRS measures different aspects of PD (mental impairment, disability, motor impairment) and it assesses the current motor status of patients through clinical observation of determined movements, providing comprehensive information on PD patients’ impairments to understand their actual damages [2].

In the early stage of the disease, PD patients often experience swallowing difficulties, and vocal disturbances such as dysphonia, monotonia and hypophonia. Therefore, voice degradation is considered an initial symptom, although this may not be evident [3]. There is a connection between voice disturbances and the onset of PD. Speech is one of the most complex motor actions in terms of control and, therefore, it may be susceptible to mild degenerative changes in the dopamine supply circuits affected in the pathophysiology of PD [4]. The corresponding symptoms that occur in the muscles of the body (tremors, rigidity, and slowness of movement) can also manifest in those involved in speech and swallowing. Speech and voice changes can be considered as detectable prodromal markers in PD using certain acoustic measures. Thus, speech is not only a carrier of relevant information for the differential diagnosis of PD but also the extraction of features of interest from voice signals for the analysis and development of recognition programs, as well as systems for diagnosing and monitoring the dynamics of the disease in patients.

PD most often affects the phonatory structure, making its impact on speech, hence the need for more objective voice measurement. Since the phonatory system affects the tension of the laryngeal muscles, it is connected to the vibratory rhythm of the vocal folds and, consequently, to the fundamental frequency. To evaluate phonation, fundamental frequency measurements are usually taken: jitter, shimmer, and harmonics noise to ratio. These measurements are not only associated to the voice production systems but also to the physiological correlates that come from the symptomatology of PD [5]. Among the findings in the voice’s evaluation of patients with this disease, in [6], it is estimated that between 60 and 80 percent of patients with PD present voice alterations, characterized by changes in frequency, duration, and intensity. Often, these alterations are confused with the natural changes of older adults concerning presbyphonia or depressive states. Patients with PD often reflect a decreased ability to maintain a fixed position of the laryngeal muscles, used in the sustained pronunciation of vowels, where a reduced skill to produce prosody is also observed [6].

Therefore, diagnosis and follow-up systems based on vocal alterations have been recurrently used in studies to detect and monitor the evolution of PD. However, in scales such as the UPDRS, there are elements of personal judgment and subjectivity, the latter related to aspects such as: mood, experience, fatigue. At the same time, they are prone to measurement error. All this leads to uncertainty in critical features, thus it is desirable to have a fine-tuned measure of UPDRS that allows for a more accurate assessment and follow-up of PD patients. Here, systems based on Artificial Intelligence (AI) and Machine Learning (ML) could potentially help to monitor patients. Using novel tools, it is possible to automatize some processes and apply them remotely, acting as an important support for healthcare personnel. Ultimately, leading to more effective follow up based on the analysis of increasing volumes of clinical information. Adopting ML in the medical and healthcare systems also encourages hospitals and practitioners to employ innovative strategies, procedures, and systems. For instance, the way examinations are performed, or when resources are being allocated and managed.

This work aims to propose a SVR approach to model the dynamics of UPDRS using voice features, to have a better assessment of PD patients. We introduced a mixture of kernels, in particular, a product between a local and a global version, which allows to decompose the effect and contribution of the proximal information (neighbor points) plus a general trend to the modeling of UPDRS. The improvement is quantitative and visually revealed by positioning the predictions over time for each patient. This matches with the reported evolution of those suffering with this disease. The potential benefit of these emerging indicators when analyzing and monitoring the PD from speech lies in their easy identification and interpretation.

As a result, we found acoustic voice analysis may be compelling clinical relevance, since it identifies sensitive markers of early voice impairment before dysphonia and other alterations are perceptible to the human ear, both in the early stages following diagnosis and in the advanced stages of the PD. Speech impairments are often an early symptom of the condition. Thus, modeling by SVR through speech features might be a promising technique for diagnostic support and monitoring the underlying disorder.

The outline of this article is as follows. In Section 2 we give a literature review considering the first contributions and then analyzing recent studies on this research topic. Section 3 is dedicated to introduce the data set and methodology. Also the SVR technique is described in this section. In Section 4 we describe our proposal as a combination of kernel functions to solve the support vector problem in a regression framework. Section 5 propose an implementation used in the mixed kernel SVR model. In Section 6, the results obtained in a learning scheme are shown. Section 7 contains a discussion of the findings and the conclusion appears in Section 8.

## 2 Literature review and background

Multiple studies suggest that analysis of voice tracing provides insights for early diagnosis and monitoring of the evolution of PD [7–10]. From the work of articles [11, 12] there is a record of the data set being widely used in the literature. This has allowed implementing Parkinson’s telemonitoring and telediagnostic systems based on speech signal analysis to emerge, which are more economical than other programs, as well as convenient to use. These schemes include, for example, the computer-based home analysis device (AHTD) in the early stage [11] and the telemonitoring device for recording speech signals, focused on tracking the progression of PD with the help of features extracted by signal processing techniques applied to a huge data set of 5875 speech samples from PD patients. Their goal was to estimate the unified Parkinson’s disease rating scale (UPDRS) using linear and non-linear regression approaches.

Furthermore, it is also possible to consider the use of speech pattern analysis applications and automatize learning techniques. This could facilitate the early detection, characterization, and monitoring of different neurological disorders that manifest themselves through the voice, with primary attention to PD. Regarding evaluation systems with summaries of the voice recordings, in the collection of articles [8, 13, 14] different proposals are conducted. In [8] is evaluated the relevance and correlation between features and PD score. They used a mutual information-based selection algorithm with a permutation test, feeding the data with features selected through maximum relevance-minimum redundancy (mRMR) in an SVM classifier. Although in most studies, models are evaluated through cross-validation or similar techniques, it is often necessary to consider that the data have different recordings of the same individual. Thus, to avoid bias, in [13], the Leave-One-Subject-Out approach is introduced where all voice samples from each individual were aggregated using central tendency and dispersion metrics such as median, mean, standard deviation, trimmed mean, interquartile range, and mean absolute deviation. In particular, using recordings of sustained vowels provides more information than words or short phrases. For this reason, they train their models on sets of representative terms comprising of sustained vowels, words, numbers, and short phrases. Recently in [14] original features are expressed based on a *Q*-wavelet transform, which yields more information on the covariates and improves some aspects of classifier performance.

Given the nature of repeated voice recordings and clinical aspects of the underlying dynamics of the disease, the contributions in the articles by Naranjo and others [15–17], are also worth mentioning. Based on their studies, that allowed them to collect data related to voice involvement in PD patients, they match the evolution of UPDRS with the fact that PD, as a neurodegenerative disease, reflects a certain monotony. Therefore, in [17], they suggest including non-decreasing processes, to consider the dependent structure of the data in a design based on more elaborate ideas, from which appropriate classification and modeling proposals are suggested. The relevance of their results indicates PD has a significant impact on certain vocal parameters and also identifies a non-linear relationship between UPDRS and these voice features.

Some efforts have also addressed the possibility of establishing a statistical model to relate speech parameters and UPDRS. Data on the vocal characteristics of Parkinson’s patients are diverse, some of them based on classical measures: jitter, shimmer, noise-to-harmonic ratio (NHR), and harmonic-to-noise ratio (HNR), and others based on the theory of non-linear dynamical systems, such as recurrence period density entropy (RPDE), detrended fluctuation analysis (DFA) and pitch period entropy (PPE). In practice, motor UPDRS and total UPDRS scores gathered during three- and six-month test periods have been used, even though speech recordings were collected at weekly intervals. That is, motor UPDRS, as well as total UPDRS at almost all time points, are approximations obtained by linear interpolation [12]. Therefore, they contain intrinsic noise, which adds to the natural variability of their derivation. In [18], the performance of various regression methods for monitoring and forecasting the progression of PD is compared. In [19], the use of an intelligent genetic programming-based system for the prediction of UPDRS assessment, using only data derived from simple, self-administered, noninvasive speech tests is investigated. Their system is termed geometric semantic genetic programming and is based on novelty-developed semantic genetic operators. Recently, in [20] is considered a GMM-UBM approach to assess the disease progression per speaker, introducing a model capable of representing arbitrary probabilistic densities. In speech processing, these are suitable for representing the distribution of feature vectors extracted from several speakers, given as a parametric probabilistic model using a weighted sum of *M* Gaussian densities.

Regarding PD detection, the studies [10, 21], and (2019) [22] provide a comprehensive overview. Combinations of computational approaches such as genetic algorithms and feature selection in SVM are examined. From importance measures given by the support vectors, they reduce the problems and then apply a classification scheme between diseased and control (healthy) patients. Note that the data set they employed remains as designed in (2009) [7]. To address the non-linearity and high dimensionality challenges, in [23, 24] is presented the idea of SVM kernel mixtures. In [23] is introduced a particle swarm optimization approach in a specific application of reliability-based design for the study of vehicle-weight reduction. In [24] a mixed-function-based SVR model is implemented to evaluate the Sobol indices relying on the theory of mechanical systems. A particle swarm optimization is also used along with the use of a combination of radial-based kernels plus an orthogonal polynomial kernel (Legendre’s kernel).

As we can see, schemes for parameterization and classification of voice measures have emerged, aimed at developing systems for the diagnosis and objective assessment of PD. Although robust, noninvasive early biomarkers are not yet available, it has been identified for decades that voice and speech are affected even in the prodromal stages of PD. Further research is underway to develop biomarkers based on digital signal processing and statistical learning techniques to facilitate early detection, characterization, and monitoring of different neurological disorders that manifest through the voice. Thus, diagnostic systems based on vocal disorders are at the forefront of recent PD detection studies.

The interest of our proposal goes along with two recent articles, also aiming at predicting the UPDRS using the same dataset. Although SVR is applied there to estimate disease progression values, the difference is essentially that they use clustering-based approaches in a previous stage. In [25], a neural network and self-organizing map (SOM) clustering are used to improve the accuracy and scalability of the predictions. By normalizing the data and using dimensionality reduction techniques, they integrate the outputs through SVR. Alternatively, in [26] multi-collinearity is detected, thus PCA and the EM algorithm are used to find clusters, afterwards, SVR is combined with the adaptive neuro-fuzzy inference system (ANFIS) implementation. In both cases, advantages over other methodologies are found and considering the similarity with our approach, in the section 6 we compare selected performance measures.

The major difference between our approach and the preceding methods lies in the use of two theoretically admissible kernels. This is not exhaustively explored in the literature. The gain is a better specification of the problem of monitoring and tracking the PD disease in each patient while using as much information as possible in a learning system.

## 3 Material and methods

In this section, the data set considered and the Support Vector Regression (SVR) approach are described.

### 3.1 Parkinson’s telemonitoring data set

In this article, we use the Parkinson’s Telemonitoring data set, introduced by Athanasios Tsanas and Max Little (University of Oxford) [7, 12], in a study conducted jointly by ten U.S. medical centers and Intel Corporation, who developed a telemonitoring device to record speech signals. This data set, available in the UCI Machine Learning Repository (For details, please visit the web respository page https://archive.ics.uci.edu/ml/datasets/parkinsons+telemonitoring), comprises voice recordings from 42 patients with early Parkinson’s disease recruited for a six-month trial to record speech signals for remote monitoring of symptom progression in their homes. A total of 5875 speech recordings are held from these individuals with PD, whose diagnosis occurred in the five years prior to the start of the trial and with at least two of the following symptoms: resting tremor, bradykinesia, or rigidity, with no evidence of other forms of parkinsonism. Patients ranged in age from 36 to 85 years.

After collecting these samples from people with early stage PD, the voice signals were transformed into a series of 16 biomedical voice measurements for the design of a Parkinson’s telemonitoring device. The data set contains 22 variables, 16 of which refer to voice characteristics (same as described in detail in [12]), including several measures of the fundamental frequency, various measures of amplitude variation, noise-harmonic and harmonic-to-noise ratios, non-linear dynamic complexity measure, fractal scale exponent of the signal, and pitch period entropy. PD patients were monitored for a period of six months and remained medication-free for the duration of the study [12]. Voice recordings of the subjects were obtained at weekly intervals during the study, while motor and total UPDRS were assessed only three times by the attending medical personnel: at baseline (start of the trial), and after three and six months. Missing UPDRS estimates, corresponding to weekly voice recordings, were obtained via linear interpolation. The UPDRS motor score of PD patients monitored in this study ranged from 5 to 40, whereas the UPDRS total score from 7 to 55. The focus of the transformed data is to predict motor or total UPDRS scores from voice features and to analyze the presence and severity of PD symptoms from a variety of voice features.

**Fig 1:**
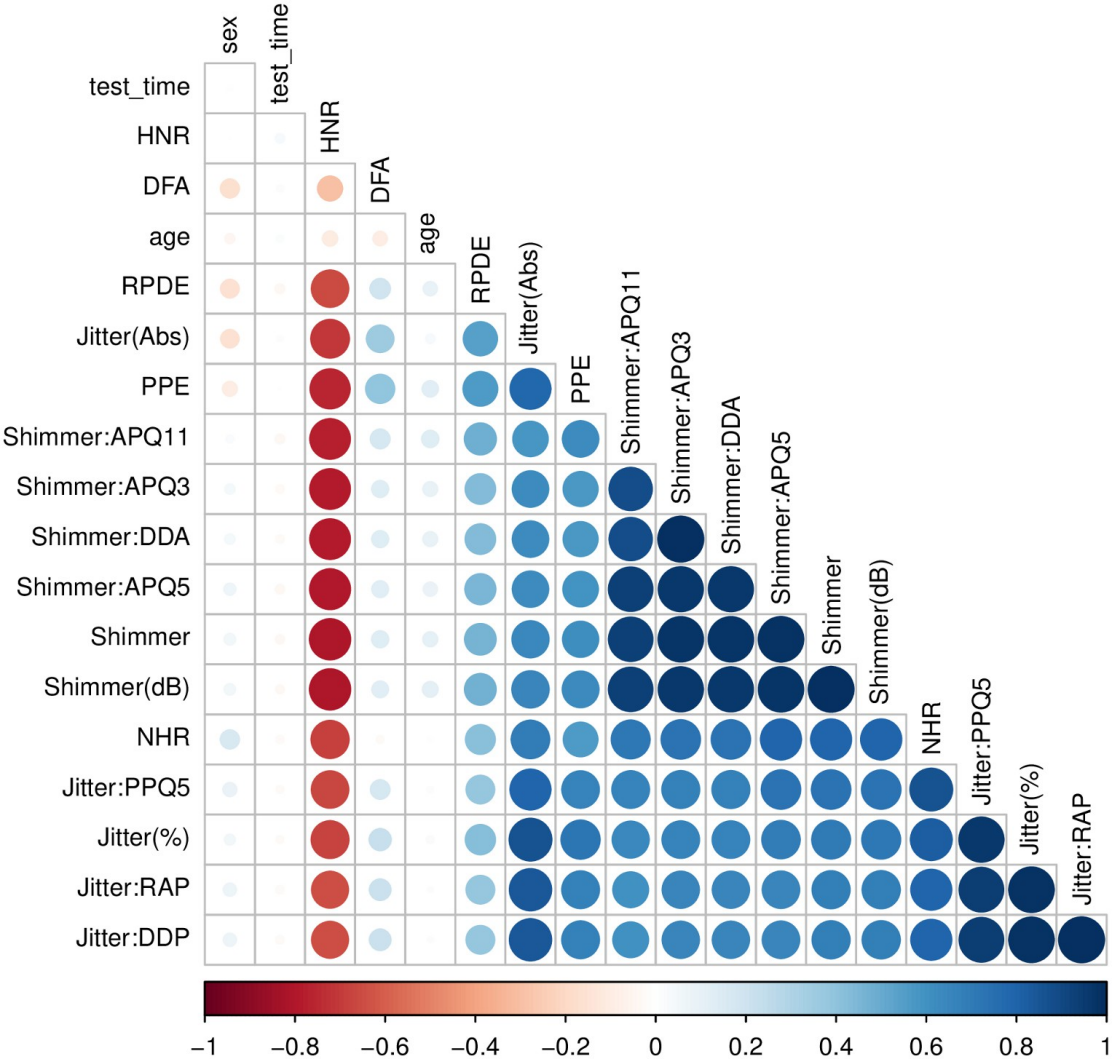
Feature correlation plot.

Table 1 shows a descriptive statistical analysis for this data set, without the gender variable (for details, see [25, 26]). The correlation plot (Fig 1), excluding the explanatory variable (Total UPDRS) points to collinearity. This phenomenon occurs in groups of variables with common characteristics, e.g. shimmer and jitter variables. Additionally we observe that HNR is related to most features. This aspect affects numerically the inference for regression methods, unlike SVR, which used the kernel trick, transforming the operations to a higher dimension feature space, which is possibly infinite-dimensional.

**Table 1:** Descriptive statistics.

Feature	Mean	Median	SD
Age	64.80	65.00	8.82
Test time	92.86	91.52	53.45
Jitter	0.01	0.00	0.01
Jitter.Abs.	0.00	0.00	0.00
Jitter.RAP	0.00	0.00	0.00
Jitter.PPQ5	0.00	0.00	0.00
Jitter.DDP	0.01	0.01	0.01
Shimmer	0.03	0.03	0.03
Shimmer.dB.	0.31	0.25	0.23
Shimmer.APQ3	0.02	0.01	0.01
Shimmer.APQ5	0.02	0.02	0.02
Shimmer.APQ11	0.03	0.02	0.02
Shimmer.DDA	0.05	0.04	0.04
NHR	0.03	0.02	0.06
HNR	21.68	21.92	4.29
RPDE	0.54	0.54	0.10
DFA	0.65	0.64	0.07
PPE	0.22	0.21	0.09
Total UPDRS	29.02	27.58	10.70

A step-by-step description of the proposed mixed kernel SVR method is given in section 5.

### 3.2 Support Vector Regression

Support Vector Machines (SVM) is a learning method for classification and regression, first introduced in [27]. When the training data set includes predictors ***x***_*i*_ and (real) observed response values *y*_*i*_, the goal of the *ϵ*-insensitive Support Vector Regression (SVR) is to find a function *f*, as flat as possible such that *f*(***x***_*i*_) deviates from *y*_*i*_ by a value no greater than *ϵ* for each training point ***x***_*i*_. Thus, the aim is to identify a surface that represents the relationship between a set of features and a target output variable (response).

The SVR approach focuses on finding the functional relationship that approximates the mapping of an input into a training set. It is a generalization of the classification problem that returns a numerical (real) output. Here, points within a band that define a fitting surface are considered. The best-fitting surface is the one that contains the most points in its band. In its formulation it tries to optimize the fit between the hyperplane or surface (understood as the prediction) and the actual value of the output, subject to a constraint, given in terms of *ϵ*-region. The latter is a band (usually symmetrical) associated with a loss function, known as a sensitivity band. The *ϵ*-region penalizes observations far from the output *y*_*i*_. Being a tuning parameter, it is known that small values of *ϵ* assign little error tolerance. Hence, a larger number of support vectors may be required in the fitting. The support vectors are the data points nearest to the optimal hyperplane, such hyperplane remains unchanged if we remove all training data points except support vectors. Larger *ϵ* values give higher error tolerance. Here, we will have a sparse solution.

In the linear case, we want to construct a margin or tolerance band for the approximation function *f*(***x***) = ***w***^*t*^***x*** + *b*, from which we will have observations inside and outside the margins. To account for training errors, slack variables $\xi_{i},\xi_{i}^{*}$ and an *ϵ*-insensitive loss function are introduced. These slack variables determine how many points can be tolerated outside the tube and have zero values for points inside the tube, however, their values increase for points outside the tube according to the loss function used. Several loss functions can be adopted, such as linear, quadratic and Huber. The Huber loss function is smoother than the linear and quadratic functions, but penalizes all deviations from the desired response variable, with a greater penalty as the error increases. In practice, symmetric and convex loss functions are the most commonly used.

Using only the observations outside the margins, the slack variables are calculated and the objective function to be minimized is formulated, also known as the primal formula:

$$\begin{array}{c} \begin{array}{cc} \underset{w,b,\xi ,\xi^{*}}{\text{minimize}} & \frac{1}{2}{∥w∥}^{2}+C\sum_{i=1}^{n}(\xi_{i}+\xi_{i}^{*})\;\\ \text{subject}\;\text{to} & y_{i}-w^{t}x_{i}-b\leq \epsilon +\xi_{i},\;\;\;i=1,\ldots ,n,\\ & w^{t}x_{i}+b-y_{i}\leq \epsilon +\xi_{i}^{*},\;\;\;i=1,\ldots ,n,\\ & \xi_{i},\xi_{i}^{*}\geq 0,\;\;\;i=1,\ldots ,n, \end{array} \end{array}$$ (1)

where ***w*** is the vector associated to the fitted hyperplane (excluding the offset), the constant *C* is the box constraint, a positive numeric value that controls the penalty imposed on observations that lie outside the (*ϵ*) margin and helps to prevent overfitting (regularization). This value determines the trade-off between the flatness of *f*(***x***) and the amount up to which deviations larger than *ϵ* are tolerated.

The optimization problem previously described is computationally simpler to solve in its Lagrange dual formulation. The solution to the dual problem provides a lower bound to the solution of the primal (minimization) problem. The optimal values of the primal and dual problems need not be equal, and the difference is called the duality gap. But when the problem is convex and satisfies a constraint qualification condition, the value of the optimal solution to the primal problem is given by the solution of the dual problem.

The minimum of the dual representation is found based on Karush-Kuhn-Tucker (KKT) conditions [28]. These complementarity conditions are optimization constraints required to obtain optimal solutions and indicate that all observations strictly inside the *ϵ* tube have Lagrange multipliers *α*_*i*_ = 0 and $\alpha_{i}^{*}=0$. If either *α*_*i*_ or $\alpha_{i}^{*}$ is not zero, then the corresponding observation is called a **support vector**.

Many regression problems cannot adequately be described using a linear model. For non-linear functions, the data can be shifted into a higher dimensional space, in such a case, the Lagrange dual formulation allows the previously-described technique to be extended to non-linear functions. A non-linear SVR model replaces the inner product $x_{i}^{t}x_{j}$ with a non-linear kernel function *K*(***x***_*i*_, ***x***_*j*_) = 〈*ϕ*(***x***_*i*_), *ϕ*(***x***_*j*_)〉, where *ϕ*(⋅) is a transformation that maps the data from the input space into a high-dimensional space, called feature space. It is considered as a non-parametric technique because it relies on kernel functions.

Given a dual objective function, the non-linear SVR allows us to find the coefficients that optimize the following setting:

$$\begin{array}{c} \begin{array}{cc} \underset{\alpha ,\alpha^{*}}{\text{maximize}} & \sum_{i=1}^{n}y_{i}(\alpha_{i}^{*}-\alpha_{i})-\epsilon \sum_{i=1}^{n}(\alpha_{i}+\alpha_{i}^{*})\\ & -\frac{1}{2}\sum_{i,j=1}^{n}(\alpha_{i}-\alpha_{i}^{*})(\alpha_{j}-\alpha_{j}^{*})K\left(x_{i},x_{j}\right)\\ \text{subject}\;\text{to} & \sum_{i}(\alpha_{i}^{*}-\alpha_{i})=0,\\ & 0\leq \alpha_{i}\leq C,\;0\leq \alpha_{i}^{*}\leq C,\;\;\;\text{for}\;i=1,\ldots ,n. \end{array} \end{array}$$ (2)

Thus, the approximation function used to predict new values is:

$$\begin{array}{c} f\left(x\right)=\sum_{i=1}^{N_{sv}}(\alpha_{i}^{*}-\alpha_{i})K\left(x_{i},x\right),\;\alpha_{i},\alpha_{i}^{*}\in \left[0,C\right], \end{array}$$ (3)

where *K*(***x***_*i*_, ***x***) = *ϕ*(***x***_*i*_) ⋅ *ϕ*(***x***).

SVR attempts to provide the best fit representing the dynamics of the set of points while minimizing some prediction error. It is restricted to a constraint band centered on the surface, given in terms of the response and the fitted function. As in SVM for classification, it contemplates the use of slack variables to penalize points far from the band. In particular, points within the fitting band are of interest, where the purpose is to capture as many points as possible. Again, the constraint involves minimizing the error between the predicted response of the function for a given value of the input and the actual value of the output. It is flexible, in the sense that an ±*ϵ* deviation is allowed between them.

Loss functions of the *ϵ*-insensitive form are also adopted. That is, they penalize predictions that are farther that an *ϵ* distance from the desired output. As mentioned, the *ϵ* value determines the bandwidth. The parameter *C* is interpreted as a misclassification cost and penalizes the tolerance for points outside the *ϵ*-tube. As it approaches zero, the tolerance fades to the (unreachable) limit of a perfect fit. As with other learning algorithms, the optimal *C* or a suitable candidate can be found by performing a grid search via tuning. This type of regression is said to be robust, since it is less susceptible to noisy points (contrary to other regression approaches). The slack variables allow quantifying the two possible errors (positive or negative) for each observation. They are defined as zero for points inside the band and their value increases for points outside, according to the chosen function.

The rationale behind the choice of a SVR modelling is the fact that some interactions between covariates and the UPDRS are in fact non-linear. According to published research on voice-based PD analysis [29], the set of vocal features widely used are as follows: jitter, shimmer, shudder, harmonization of fundamental frequency parameters, recurrence period density entropy (RPDE), trend fluctuation analysis (DFA), and pitch period entropy (PPE); which all are closely related to the dynamics of UPDRS. Although the relationship between some covariates is in fact linear, it turns out that the interaction between these covariates with UPDRS is expressed in a complex fashion. Since features are given in a similar way, that is, the internal structure of selected groups of variables is equivalent, then multicollinearity exists. Thus, it has been observed that regression proposals or the use of regression trees (CART) are not appropriate.

## 4 Mixed kernel SVR

In this section, we present a proposal addressing a SVR approach based on a mixture of kernel functions. We introduce the sum and the product of the kernel functions to capture the global and local dependencies of the response variable given by the total UPDRS.

Considering the kernel trick, SVM maps the input space into a feature space through a kernel function, thereby a non-linear problem in the input space can be transformed into a linear problem in the feature space. When SVMs are used for solving classification and regression problems [30], kernel selection is an important aspect. Dissimilar kernel functions induce different SVMs settings with different performance measures. In particular, kernel selection strongly affects the solution of the regression problem [31]. Therefore, a kernel selection and parameter selection strategy are selected, by studying kernel mixtures and their application in Support Vector Regression.

It is important to observe that a kernel function can be interpreted as a measure of similarity. That is, the kernel map *K*(***x***, ***z***) is large when ***x*** and ***y*** are similar. This motivates the design of kernels for a specific data type or application, since prior knowledge might suggest a similarity measure relevant within a given context. Hence, picking a kernel according to the domain expertise or intuition of the observations’ geometry seems more appropriate.

The following holds for any ***x***, ***z***:

$$K\left(x,z\right)=\frac{1}{2}[{∥\phi \left(x\right)∥}^{2}+{∥\phi \left(z\right)∥}^{2}-d{\left(\phi \left(x\right),\phi \left(z\right)\right)}^{2}].$$

Thus, *K*(***x***, ***z***) measures the similarity between ***x*** and ***z*** as the opposite of the square distance *d*(*ϕ*(***x***), *ϕ*(***z***))^2^ between their images, up to square of their norms. This notion is useful in designing kernels and in understanding kernel methods. Here, the first and basic condition that a kernel function is required to fulfill to be admissible is the Mercer condition:

**Theorem 1 (Mercer)**. *Under certain technical conditions (see* [31]), *if*

$$\int_{X\times X}K\left(x,z\right)f\left(x\right)f\left(z\right)dxdz\geq 0,\forall f\in L_{2}\left(X\right)$$

*holds, then each positive definite kernel K*(***x***, ***z***) *defined on a compact domain* X×X *can be written in the form*

$$K\left(x,z\right)=\sum_{i=1}^{M}\lambda_{i}\phi_{i}\left(x\right)\phi_{i}\left(z\right),M\leq \infty ,$$

i.e. it is possible to write *K*(***x***, ***z***) as an inner product in some feature space.

The contribution of this work is the search for kernel functions performing under a combination, to detect effects different from those typically encountered by kernel functions. The proposals [32, 33] appear in the context of classification. Theoretically, the following results are supported by Smola and Schölkopf (2004) [31], Corollary 3 and Theorem 5. The following functions are admissible kernel functions:

1. *K*(***x***, ***z***) = *K*_1_(***x***, ***y***) + *K*_2_(***x***, ***z***)
2.
$K\left(x,z\right)=\beta K_{3}\left(x,z\right),\beta \in R$

3. *K*(***x***, ***z***) = *K*_1_(***x***, ***z***) ⋅ *K*_2_(***x***, ***z***)

In summary, the key aspect to emphasize is that both positive linear combinations and the product of kernels are also admissible kernels.

According to [32, 33], kernel functions may be categorized as the global and local kernel functions. For global kernel functions, points far away from the test point have a large effect on the kernel values and their generalization ability is said to be strong if the training learning ability does not overfit. In contrast, the local kernel function has a strong learning ability in training, yet its generalization ability becomes weak since only points close to the test point have a substantial effect on the kernel values. Given their respective characteristics of the global and local kernel functions, if the two type of kernel functions are mixed into a hybrid kernel function, we will be able to achieve a satisfactory classification performance.

As an example of a global kernel, one can find the radial basis functions (RBF), which have attracted widespread interest in the literature. Gaussian kernels are the most widely used and have been studied in great detail in related fields. For our purposes, we will express it as:

$$\begin{array}{c} K_{RB}\left(x,z\right)={\text{exp}(\gamma ∥x-z∥}^{2}). \end{array}$$ (4)

Using the Gaussian radial basis kernel provides a high learning capability, by tuning the hyperparameter *γ*, because only one function is available for near training points, however, its generalization could be weak.

An attractive option for local kernel functions are polynomials, as represented by the following formula:

$$\begin{array}{c} K_{P}\left(x,z\right)={\left(1+\sigma \left\langle x,z\right\rangle \right)}^{p}, \end{array}$$ (5)

where the power *p* is the polynomial degree governing the flexibility of the approximating function and *σ* is a hyperparameter related to generalization ability. The polynomial kernel has good generalization ability in the appropriate parameters since it considers the near test point far across the data points. Higher degree kernels produce a more flexible representation, although it tends to have higher complexity and overfitting.

Considering the global and local kernel functions characteristics, an alternative is to merge both types of kernel functions into one kernel function bringing together both properties. Therefore, if the polynomial function and the Gauss Radial Basis function are mixed to generate a new multiple kernel function, which could have better learning and generalization abilities. In their respective papers, the authors propose hybrid kernels as a linear combination. Recall, if *K*_1_ and *K*_2_ are two kernels, then any linear combination, *β*_1_*K*_1_ + *β*_2_*K*_2_, with *β*_1_, *β*_2_ ≥ 0, is an admissible kernel.

In this paper we mainly analyze the choice of two mixed kernels based on the idea of capturing global and local changes. It is known that, in the polynomial kernel, items distant from the test point have a large effect on the kernel values. Whereas, in the radial-based kernel, points close to the reference point have a large effect on the kernel. Two cases will be considered:

1. A convex linear combination of a radial basis kernel and a polynomial kernel.
2. The product of radial and polynomial basis kernel functions.

In both cases, such parameters require tuning by the usual learning techniques.

Based on the above ideas, a mixed (sum) of kernel functions is initially proposed:

$$\begin{array}{c} K_{sum}\left(x,z\right)=\beta K_{RB}\left(x,z\right)+\left(1-\beta \right)K_{P}\left(x,z\right),\beta \in \left(0,1\right). \end{array}$$ (6)

The following sub-section explains the choice of the *β* parameter. Also, knowing that if *K*_1_ and *K*_2_ are two kernels, then *K*(***x***, ***z***) = *K*_1_(***x***, ***z***)*K*_2_(***x***, ***z***) is an admissible kernel. A second proposal is possible to establish as:

$$\begin{array}{c} K_{prod}\left(x,z\right)=K_{RB}\left(x,z\right)\cdot K_{P}\left(x,z\right). \end{array}$$ (7)

In this way, it is possible to create functions from transformations and products of kernel functions. In addition, in the following subsection, we discuss our SVR implementation.

## 5 SVR implementation

The proposal procedure can be summarized in the following steps.

**Mixed kernel SVR**:

1. Import data set and extract the features variables and the response separately.
2. Divide the data set into train and test samples.
3. Select the kernel combination to capture mixing effects in the dynamics of the response. For example, in R statistical software one can customize the kernel function using the kernlab library.
4. Initialize the SVR model. In a previous step it is suggested to fit individual kernel functions and then use a combination which yields an admissible kernel through some specific kernel operation (see section 4).
5. Tune and determine the hyperparameters involved in the model. Generally, optimal choices of *C*-cost, *ϵ* bandwidth and proper kernel function parameters need to be set.
6. Fit the SVR model (see Eqs 2 and 3). As in a supervised-learning environment, it is common to obtain a class of models and choose the most appropriate according to the goal problem.
7. Perform SVR model predictions on the train and test sets.
8. Calculate the error between the actual output values and the predictions to obtain the model performance evaluation.
9. Finalize and report performance metrics. For example, we use MAE, RMSE and R^2^, yet other regression model metrics can be used.

In our application, the aim is to explain UPDRS for each patient based on a set of explanatory variables, such as voice signal measures (The programs for this article were generated using R software with the support of the following packages: e1071, caret and kernlab. Program code will be available at https://github.com/rbarcenas). To achieve this, we worked in a SVR setting, which considers the points that are within a band around the decision function. The best fit line is the hyperplane that has a maximum number of points. A total of 5875 data observations are available from 42 Parkinson’s patients (28 men and 14 women) where speech measures were recorded at different times, with repetitions. This means that different voice samples can be taken at the same point in time. On average, 140 recordings are available for each patient. We fit an *ϵ*-insensitive SVR and, in view that the choice of the kernel function is one of the most important decisions, three settings were examined. First, using just a radial basis kernel. Then a mixture kernel was considered as a sum of a radial basis kernel and a polynomial kernel. Finally, a mixture given by the product of the radial and polynomial kernels is also included.

We splitted the set of observations into training and test sets, which is common practice in the learning domain. In addition, a disjoint training set of approximately 8% of the total (476 randomly chosen observations) is taken for initial parameter tuning. Then, from the rest, 4223 observations (approximately 72% of the total) are used in the SVR training set. As a result, the total number of test samples is 1176 (20% of the total) used to verify the performance of the learning algorithm on data unused for fitting. We have made an effort to ensure that each patient has the equivalent proportion of observations in training and testing, to represent a corresponding number of replications in each subsample.

To improve the performance of SVR, we will need to select the best parameters for the model in a hyperparameter optimization process. The selection of the parameter *ϵ* of SVR determines the level of accuracy of the fit, when we choose small values, the identification is less flexible. The algorithm is trying to approximate the objective function as well as possible, even though if some points are still outside the confidence band delimited by *ϵ*. Therefore, we have to contemplate the possibility that some of the errors are larger than *ϵ*.

In SVM, the penalty term is denoted as *C* which is used as a regularization hyperparameter referred to as tolerance degree or cost, likewise defined for linear and non-linear solutions. As *C* increases, the tolerance for points outside *ϵ* also increases. A high value of *C* results in an increased penalty to the algorithm when it classifies poorly, but at the same time, it may cause overfitting of the training data. If *C* is small, the penalization for points far from the approximating function is low, allowing a tight hyperplane with a large margin at the cost of a larger number of far points. As *C* approaches 0, the tolerance is close to 0 and the solution might become infeasible.

First, we selected an initial subset with 476 data points and using the tune method based on a grid search over *ϵ* and *C*, with the values *ϵ* = 0, 0.1, 0.2, …, 1, and cost *C* = 2^2^, 2^3^, 2^4^, …, 2^9^, we use the optimal solution train the algorithms. Also, since the approximation level is defined according to the selection of the parameters, after they are optimized we obtain a better result.

As technical remark, it is not common to use resampling or cross-validation because of the nature of the SVM solution, instead the Sequential Minimal Optimization algorithm is often chosen for solving the SVM QP problem and most SVM formulations. Next, we performed a comparison with other approaches widely used in classification and regression: Classification and Regression Trees (CART) using a regression tree, a *K*-nearest neighbors algorithm, variants of boosting (XGBoost, and GLM Boost), a simple Neural Network, Random Forest and Gradient Boosting Machines. Recall that Random Forests (RF) are based on the idea of Bagging from regression trees. Next, XGBoost, and Generalizaed Linear Models by Boosting, and Gradient Boosting Machines (GBM), are different versions of boosting, typically used for classification, although here it is employed in a regression setting. For these algorithms, the same splitting of the test and training sets is used. Furthermore, the parameters were optimally tuned. Due to how these methods are trained, a process of resampling (*K*th-fold cross-validation) is involved.

The selection of the *β* parameter (Eq 6) involved a grid search by specifying many values to be tested in the convex linear combination. The idea behind testing all combinations is to test them and choose a combination giving the best possible performance. Here we chose the value *β* = 0.8. It implies a higher weight for the local (radial-based) kernel than for the polynomial kernel, whose coefficient was 1 − *β* = 0.2.

To compare model performance we consider three performance metrics: the Mean Absolute Error (MAE), the Mean Squared Error (MSE) and the R-squared (R^2^) as a way to evaluate the fit.

Consider each *y*_*i*_ value with the associated predicted value ${\hat{y}}_{i}$ and see how far away they are with a simple difference. Note that the expression ${\hat{y}}_{i}-y_{i}$ is the error, if we make a perfect prediction ${\hat{y}}_{i}$ will be equal to *y*_*i*_ and the error will be zero. In particular, we will monitor MAE given by:

$$MAE=\frac{1}{n}\sum_{i=1}^{n}\left|{\hat{y}}_{i}-y_{i}\right|.$$

Calculating the error for each data point and sum, we will have the mean absolute aggregate of the errors. Also, we consider the *R*-squared given by:

$$R^{2}=\frac{\sum_{i=1}^{n}{\left({\hat{y}}_{i}-\bar{y}\right)}^{2}}{\sum_{i=1}^{n}{\left(y_{i}-\bar{y}\right)}^{2}}.$$

A common way to measure error in ML is to use the RMSE. To compute it we use the formula:

$$RMSE=\sqrt{\frac{1}{n}\sum_{i=1}^{n}{\left({\hat{y}}_{i}-y_{i}\right)}^{2}}.$$

## 6 Results

**Fig 2:**
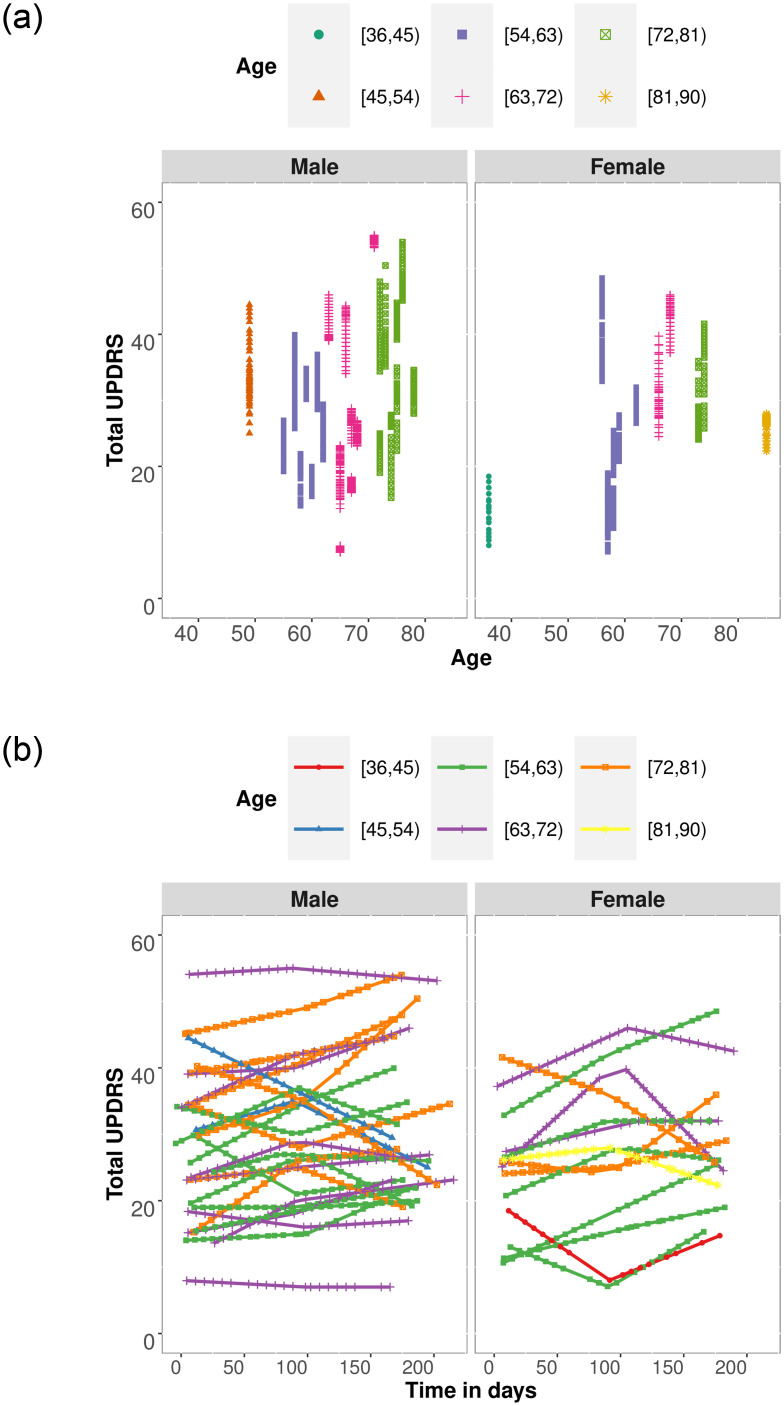
Total UPDRS PD progression by age and genre. (a) UPDRS grouped by genre and (b) UPDRS progression in time.

Grouping by gender and considering age as the horizontal axis, on the Fig 2a shows the UPDRS values for female and male patients by age, where each one is represented with a different color according to separation by age groups. The vertical dynamics of the UPDRS do not obey the chronological order of the measurements, there is an upward trend in the UPDRS measures as age increases. Although there are some exceptions. There could be more than one patient at each age point in the Fig 2a. The younger the age, the lower the variability of the UPDRS and it increases with age. In addition, aside from the imbalance in the samples, male patients are more affected, which is also observed in the comparison. For female patients, the separation is clearer. Two groups are distinguished: patients under 65 years of age and those over 65 years of age, with a UPDRS being greater than 25. From the top, note that there is a presence of higher UPDRS values for older patients. Such regularity is more visible when examined by gender, since the UPDRS values are lower for female patients than for male patients. Under this measure, the distributional summaries of UPDRS reveal this difference between both groups.

The Fig 2b shows the UPDRS values viewed over time along with the trial. It was only possible to record it at three points in time (at baseline, 3 months, and 6 months from start); all other values correspond to an interpolation. The lines connecting the points (labeled with a different color according to the age group) show the dynamics for each patient. There is heterogeneity among the curves derived from the subjectivity of the measurement. Moreover, it does not match the fact that it is a non-decreasing and slow monotonic measure. The patient with the lowest UPDRS (below 10) is 65 years old, in contrast to the one with the highest value (around 55) who is 71 years old. The median UPDRS is 27.58 while the age has a median of 65.

Table 2 shows the results for the training set. At the top, we list the metrics in the training of each algorithm, where the MAE, R^2^, and RMSE appear in each row, respectively. In the comparative, an improvement was observed as the kernel version was modified. We achieved a significant improvement by using a convex linear combination of a Radial basis kernel and a polynomial-based kernel, resulting in the values: 7.04 (MAE), 0.329 (R^2^), and 8.77 (RMSE). The best fit is obtained for the kernel mixture given by the product of radial and polynomial basis. Here, the lowest MAE equal to 4.9 is found, whereas the values of R^2^ and RMSE are 0.6243 and 6.22, respectively. Thus, this favorable result in training will be used to evaluate its predictive capacity.

**Table 2:** Training performance comparison for different algorithms.

**Training**	**CART**	**KNN**	**XGBOOST**	**GLM Boost**	**NNET**
MAE	6.724	6.545	7.119	8.145	6.574
R^2^	0.368	0.455	0.369	0.151	0.407
RMSE	8.507	8.168	8.680	9.876	8.277
**Training**	**RF**	**GBM**	**SVR Radial**	**SVR Mixed Kernel sum**	**SVR Mixed Kernel product**
MAE	6.490	7.490	7.601	7.040	**4.902**
R^2^	0.467	0.418	0.217	0.329	**0.624**
RMSE	8.160	8.920	9.460	8.770	**6.220**

In Table 3, we show the same metrics computed for testing. Each model was taken, and we performed the prediction on the test set. The prediction ability of SVR under the mixed kernel product gives the best metrics (RMSE 5.93, 0.616 R^2^ and 7.51 RMSE). Further, we notice a similar performance for the others, showing some uniformity for different methods. By considering the training results, the SVR predictions under the kernel product are acceptable and there is a reasonable trade-off in terms of modeling and generalization capability.

**Table 3:** Testing performance comparison for different algorithms.

**Testing**	**CART**	**KNN**	**XGBOOST**	**GLM Boost**	**NNET**
MAE	6.815	6.443	7.481	7.960	6.708
R^2^	0.334	0.395	0.318	0.166	0.385
RMSE	8.638	8.240	9.193	9.668	8.467
**Testing**	**RF**	**GBM**	**SVR Radial**	**SVR Mixed Kernel sum**	**SVR Mixed Kernel product**
MAE	6.050	7.420	7.440	7.170	**5.930**
R^2^	0.437	0.403	0.235	0.293	**0.616**
RMSE	8.110	8.880	9.270	8.960	**7.510**

As a last step, the graphs in the Fig 2 suggest a natural grouping by age and gender. Hence, four subgroups are proposed, dividing patients by gender and with a threshold of age equal to 65 years. As a result, we have four separate clusters, as follows: Group 1 (women under 65 years), Group 2 (men under 65 years), Group 3 (women over 65 years), and Group 4 (men over 65 years).

In Table 4 we show the results when considering the best kernel found before, reducing the number of variables by no longer using age and sex. The kernel combination is used as the product of radial and polynomial base of degree 2. Within each group, the parameters were independently tuned. From the metrics, separating by age and gender variables favors a better interpretation and modeling of the UPDRS disease progression assessment measure.

**Table 4:** SVR proposal performance: Training and testing by groups of gender and age.

**Kernel product**	**Group 1**	**Group 2**	**Group 3**	**Group 4**
Training				
MAE	2.53	1.58	1.85	1.41
R^2^	0.88	0.92	0.88	0.96
RMSE	3.70	2.65	2.27	2.19
**Kernel product**	**Group 1**	**Group 2**	**Group 3**	**Group 4**
Testing				
MAE	3.17	3.90	2.99	3.93
R^2^	0.85	0.72	0.62	0.76
RMSE	4.13	4.96	4.03	5.41

In addition, we defined a gain measure of the performance metrics MAE, R^2^, and RMSE, obtained as the percentage decrease of the same metrics concerning the best model in the fist modelling (mixed kernel product). We calculated this percentage gain for the training and test sets for each of the groups given by gender and age. For both training and test, there is a significant improve in all metrics. For training, the best performance was found in Group 4, the MAE still decreased by almost 71.22%, while the R^2^ and RMSE have an increase and decrease of 53.77% and 64.79%, respectively. Groups 2 and 3 have a similar behavior in the gain measure, being similar to Group 4. For the test set, a significant gain in prediction is also found. A positive performance appears in Group 4, with a decrease from 49% and 46% (in MAE and RMSE). However, the increasing for R^2^ is observed in Groups 1, 2 and 3, exhibiting similar gains, ranging between 18% and 39%. Hence, better predictions are expected for unobserved data.

**Fig 3:**
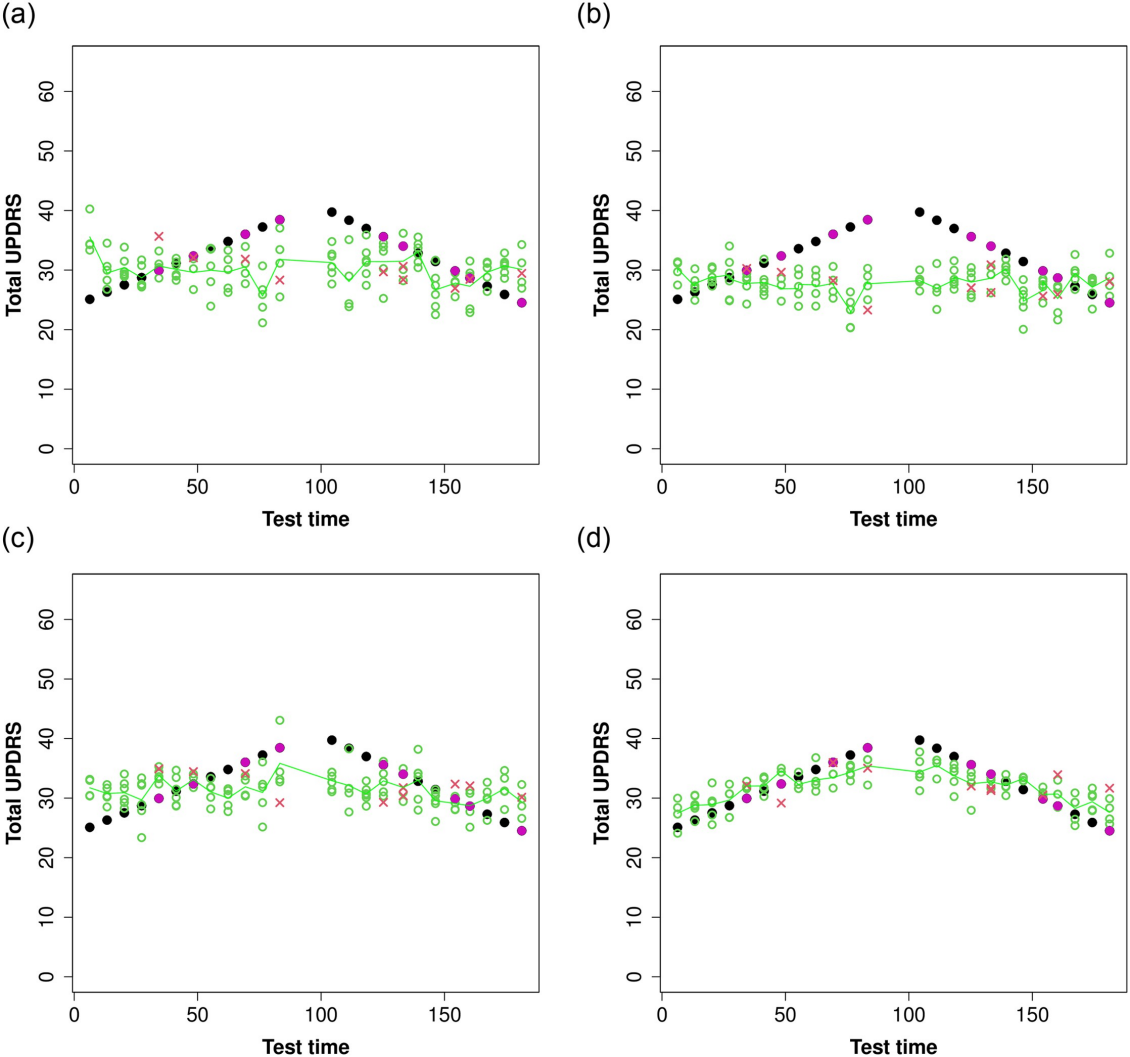
Patient fitting example: Black points are actual UPDRS values, whereas green points are the fitted values plus a smoothing (green line). Points in magenta represent selected test points with red crosses as their prediction. (a) Radial basis kernel, (b) Sum of kernels, (c) Product of kernels and (d) Product of kernels grouping by age.

For each patient, there is an improvement in the specification and explanation of the UPDRS dynamics based on the voice features. These findings can be shown in a graphical form by representing the evolution of the models. Using registered times as a reference only, since they are not included in the modeling, the graphs in Fig 3 show the UPDRS model analysis for one patient, whose dynamics is one of the most difficult to capture. Here, the different model setups are represented, changing the kernel functions at each stage. The black points are the actual UPDRS values and the green points are the fitted values for the model using the training sample plus a line of the same color, representing a smoothing of this fit. Next, solid dots in magenta are actual values from the test sample for this patient and the red crosses are the corresponding predictions. We note that, based on the actual data, the behavior is atypical, with accelerated growth and then an abnormal decay. In the upper left, we started from the SVR model with a radial basis kernel and detected a high variability in the adjusted values. There is also the difficulty of not capturing the dynamics, as the points are not able to detect the change. The predictions also deviate from the actual values.

In [25], for the best model given by the combination of EM plus PCA and SVR, they only report the values: MAE equal to 0.4431 and R-Squared as 0.991. With respect to [26], for the SVR model, they report an RMSE value equal to 1.243 for its training set and a test RMSE as 1.368. Considering an adjusted R^2^, they obtain values of 0.843 and 0.761, for the training and test sets, respectively. In our case, adopting the optimal grouping by age and gender, for the training sample, we get, on average, values of 1.8425, 2.70, and 0.91 for MAE, RMSE, and R^2^, respectively. For the test data, on average, we obtained values of 3.4975, 4.63, and 0.7375 for these measures.

This comparative analysis requires attention, as there are the other relevant differences in the data processing, namely the use of clustering and data normalization techniques. However, we can state our results are competing and also have the distinction of detecting the global and local dynamics of the UPDRS. Meanwhile, previous methods are focused on reducing the prediction error and dimension when applying clustering techniques.

When using a mixture of kernel functions, in the Fig 3b, the variability of the fitted values is reduced, although a closer approximation to the true dynamics remains to be seen. It suggests the local component, given by the basis kernel, performs adequately, decreasing the amplitude of the band in which the potential values are distributed. However, the global component in the polynomial does not reach the desired level for tracking the UPDRS behavior for this patient. Similar phenomena occur for some other patients. This is characteristic of this measure of disease progression, since, as we have mentioned, it is determined by different factors, clinical and subjective assessment, given by the specialist, and the completion of the questionnaires by the patient.

In the Fig 3c, we note a contribution made by introducing a kernel product. Here both components act locally and globally. The variability is reasonable and indicates the range of values in which the UPDRS is observed. It also gives a more detailed idea of how the UPDRS changes over time (green line). Although it goes up and down, the transition is slow and overall increasing, which reflects the disease progression. The latter is captured exactly in the last part of our analysis (see Fig 3d). Fixing the kernel combination as the product of radial and polynomial basis. Out of all kernel proposals, the latter is the best model; as the variability is acceptable, depicting an envelope of the data. The green line representing the smoothing is also a more accurate description of the dynamics, stabilizing those changes, whereas appears to be more stable than the other fits. Again, we can say there is consistency with the clinical definition and results in the dynamics as expected, plus the bonus of having a quantification of the uncertainty. We finally note the proximity of the magenta dots and red crosses, indicating a substantial improvement of the predictions.

## 7 Discussion

The implementation of additional diagnostic and telemonitoring systems based on new criteria could be very useful in the evaluation of PD. Follow-up studies of the evolution of PD with this learning tools suggest that the course of the disorder is not linear and that deterioration is variable and more accelerated in the early stages of the disorder. In each patient, progression of the disease manifests itself differently and at a different rate. Therefore, the analysis of the voice recordings helps monitor the progression of PD. Acoustic voice measurement analysis may act as an objective and non-invasive diagnostic biomarker in PD. In the future this would facilitate diagnosis and follow-up, potentially improving the quality of life of patients.

Extensive research has shown that some measures obtained from acoustic voice analysis are useful for identifying early changes that are not necessarily clinically noticeable. Such analyses serve as an objective noninvasive tool for the diagnosis and monitoring of PD. Therefore, monitoring the speech signals of PD patients could pave the way for a better understanding of disease progression and lead to enhanced diagnostic and treatment methods. Great efforts have been based on the classification of cases of patients with clinical suspicion, since there is also no determinant test to indicate the presence of the disease. Instead, in our case, we addressed the surveillance and follow-up of patients, to assess progression under a baseline response measure (UPDRS). Our study reveals besides the fact that voice signals not only convey relevant information for the differential diagnosis of PD, but it also illustrates that the analysis of features of interest has the potential to be automatized into machine learning systems by extracting different aspects related to voice dynamics.

The UPDRS as a scale for the assessment of PD through motor and non-motor symptoms, is also valuable for the assessment, follow-up and treatment of patients. We used this scale as a response variable, and we considered a set of covariates from voice recording measures to explain it. Therefore, our main objective has been to find a non-linear approximation surface, which determines the relationship of the data characteristics and the total UPDRS as a target variable. In such a way that the data points closest to the approximating hyperplane and hence to the support vectors are within that boundary line.

Regarding the difficulties involved in the diagnosis and treatment of the disease, note the following. Early detection is not always feasible, since it requires examination of neurological specialists skilled in this disorder. Thus, a telemonitoring system would help to some extent by solving the problem of detection, patient follow-up perhaps issuing recommendations, including the scheduling of appointments with specialists, thereby enabling people with the greatest difficulties inherent to the disease to quickly access the health care they need. To achieve this goal, telemonitoring schemes and intelligence systems based on statistical learning become effective in supporting PD patients to receive healthcare services in a resource-optimized landscape.

With proposals such as ours, the criteria for subjective assessment of the UPDRS level can be improved, in terms of its description and uncertainty quantification, by adopting different values and adhering to more flexible criteria, i.e., the descriptive evaluations can have a broader margin, as shown in this work, so as to constitute a truly informative measure of disease progression, covering multiple potential scenarios. Nevertheless, any decision supported on the information provided by an algorithm-based automated processing system must be made and supervised by a qualified expert, capable of evaluating a decision based on a concrete context.

In addition, it is important to point out the drawbacks of the SVR methodology with mixed kernels. By its connection with support vector machines, the complexity of the solution is of the order $O(n^{3})$ and $O(n^{2})$, with *n* the number of observations in the training set. That means solving a quadratic programming problem with several variables equal to the number of training data. Recall, the kernel is a function for converting a lower-dimensional data set to a higher-dimensional data set. A kernel aids in the search for a hyperplane in higher-dimensional space while reducing the computing cost. When the size of the data grows larger, the computing cost usually rises. Therefore, when the number of observations is very large, this problem becomes computationally expensive. Similarly, if the number of features for each data point exceeds the number of training data samples, this methods will underperform.

## 8 Conclusions

PD belongs to a group of degenerative diseases affecting predominantly the adult population. Following Alzheimer’s disease, it is the most prevalent. Its major difficulty rests in the fact that there is no definite test and it shows clear clinical signs until its advanced stages. Given the degradation of motor and non-motor capacities, as well as the slow progression that affects diverse functionalities, amongst which alteration of movement is the clearest symptom. However, other non-motor disorders also appear, such as depression, sleep disorders, voice and speech disturbances.

The evidence suggests the existence of a non-linear relationship between the selected speech signal features and the assessment given by a subjective measure, such as UPDRS. In this work, in particular, a non-standard regression approach is used. SVR is a powerful algorithm that allows us to choose the degree of error tolerance, both through an acceptable margin of error (*ϵ*) and by tuning our tolerance to fall outside that acceptable error rate. The ability to include slack variables hedges against outliers and diminishes sensitivity to extreme values. These quantities determine how many points can be tolerable falling off that *ϵ*-band. That is, the output is the line of best fit having a maximum number of points, where, as far as our application is concerned, it worked out quite accurately.

The major contribution of this work lies in proposing a SVR regression based on the combination of kernel functions, which in practice has proven to be helpful in capturing different non-linear effects from those encountered in conventional regression approaches. The novelty of our method is the idea of combining a local component, given by the radial basis kernel, and a global component through a polynomial kernel. Selecting a combination of these two achieves a satisfactory level to track the expected dynamics of the UPDRS. This allows to reduce the variability in the monitoring of the potential values of this measure of disease progression, which, as we have mentioned, is determined by different factors, clinical and subjective assessment. While the variability of the fitted values decreases, a closer approximation to the true dynamics should be observed. Thus, when compared to other machine learning approaches, our proposal achieves the best performance regarding proper regression fitting measures: Mean Absolute Error (MAE), Mean Squared Error (MSE) and R-squared (R^2^). In addition, given factors such as age and gender from data collected throughout their follow-up, revealed that our approach can better describe the dynamics of patients’ UPDRS.

Moreover, the work is supported by well-known theory, which derives important results from kernel functions, such as the cited Mercer’s Theorem and the fact that the sum and product of admissible kernels produce an admissible kernel. This allows an automatic way of modeling, with acceptable matching to the clinical criteria. The underlying variability of diagnostic and follow-up measurements is extended, managed, and interpreted. Splitting training and testing enables a consistent way to update predictions. These systems are expected to aid the follow-up of the disease. The examination of speech and voice changes and acoustic parameters associated with PD, including the non-linear effects between them, will serve for both early detection of the onset, progression, and severity of the disorder, as well potentially assess the efficacy of pharmacological and surgical treatments, rendering them particularly relevant at present and in perspective for the future. Within the specific context and application, the choice of mixed kernels has been successful for the aim, not to classify, which has already been studied before, but, given the diagnosis, to use non-invasive measures to approximate a measure with different clinical and judgmental components. Our approach provides a solid reference of the progression of an already complex condition. Since, we have validated the proposal by testing metrics as well as comparing with other existing algorithms.

AI and ML methods applied in the medical field are redefining the paradigm of the medical domain from many perspectives. It is possible to use medical systems processing vast volumes of information, which otherwise would be very difficult to analyze. Hence, AI already offers many advantages in medical sciences. New technologies are becoming essential to effectively describe some aspects of pathologies with minimal risk of error. When applied to neurology through medical systems, it is feasible to gain a understanding of such a complex entity as the brain. The result is a deeper understanding of how neuronal connections work helping in the diagnosis and treatment of patients of diseases such as Parkinson’s, Alzheimer’s, or senile dementia.
